# *Epimedium brevicornum Maxim*. Extract exhibits pigmentation by melanin biosynthesis and melanosome biogenesis/transfer

**DOI:** 10.3389/fphar.2022.963160

**Published:** 2022-09-29

**Authors:** Chen Hong, Lili Yang, Yifan Zhang, Yiming Li, Huali Wu

**Affiliations:** ^1^ Department of TCM Chemistry, School of Pharmacy, Shanghai University of Traditional Chinese Medicine, Shanghai, China; ^2^ Department of Dermatology, Shuguang Hospital Affiliated to Shanghai University of Traditional Chinese Medicine, Shanghai, China

**Keywords:** Epimedium brevicornum Maxim., melanogenesis, melanosome biogenesis, tyrosinase activity, melanogenic ingredients

## Abstract

Epimedium brevicornum Maxim. (Epimedii Folium) is a traditional medicine widely utilized in China for sexual dysfunction and osteoporosis treatment. Recently, studies have reported that Epimedium flavonoid icariin displayed hair growth and melanogenic ability by targeting tyrosinase activity. Nevertheless, icariin hydrolysate icariside II and icaritin cause depigmentation due to their tyrosinase inhibition. These pigment functional discrepancies from Epimedium constituents arouse our great interest. Then, this study focused on the pigmentation effects of Epimedii Folium extract (EFE) on melanin synthesis and melanosome biogenesis/transfer, and further identified the bioactive constituents. First, in *in vitro* systemic studies, we discovered that the potent melanogenic and repigmented effects of EFE were dependent on concentration and amount of time in multi-melanocytes, normal human skin tissue, and vitiligo perilesional areas. *In vivo*, EFE exhibited repigmented effect on two kinds of depigmented models of N-phenylthiourea-induced zebrafish and hydroquinone-induced mice. Mechanistically, EFE strongly promoted tyrosinase activity and upregulated the protein expression of tyrosinase families which finally contribute to melanin biosynthesis by activating the MAPK/ERK1/2 signal pathway. In addition, EFE effectively increased melanosome number, accelerated melanosome maturity and cytoplasmic transport through the growth/extension of melanocyte dendrites, and induced melanosome transfer from melanocyte to keratinocyte for pigmentation. The six main flavonoid ingredients were identified among EFE. Compared to others, epimedin B (EB) was confirmed as a high-content, low-toxicity, and effective melanogenic compound in EFE. Taking all these together, this study systematically demonstrates the potential pigmentation effect of Epimedium brevicornum Maxim., and clarifies its related molecular mechanisms and melanogenesis basis. These results give additional insight into Epimedium herb pharmacology and may provide a novel therapy basis for hypopigmentation disorders.

## Introduction

Epidermal melanin has important evolutionary and physiological implications, particularly for humans. Melanin pigment (racial pigmentation) can protect human skin against ultraviolet (UV)-induced skin damage using its optical and chemical filtering functions (Slominski et al., 2004). Insufficient skin pigmentation will leave the underlying tissue improperly protected from ultraviolet (UV) radiation (Costin and Hearing, 2007) and contribute to the development of hypopigmentation disorders (Rose, 2009). Melanin is produced by melanocytes which reside at the basal layer of the human epidermis, and melanosomes containing melanin are subsequently transferred to keratinocytes. In this process, various steps include melanogenesis, melanosome maturity/transportation to the cell membrane, transfer to adjacent keratinocytes through extended dendrites, and determination of skin, hair, and eye pigmentation (Lin et al., 2007).

Melanin is the main contributor to pigmentation. There are two main types of melanin—red/yellow pheomelanin and brown/black eumelanin. Both melanin are polymorphous biopolymers that are synthesized by complex multistep transformations of L-tyrosine. Hydroxylation of L-tyrosine to L-dopa is the rate-limiting step in melanin synthesis and is catalyzed by tyrosinase (TYR) which is a copper-containing membrane-bound located in melanosome (Videira et al., 2013; D'Mello et al., 2016). L-phenylalanine in the cytosol may be converted into tyrosine by phenylalanine hydroxylase (PAH), serving as the substrate for tyrosinase. Aside from tyrosinase, tyrosinase-related protein 1 (TRP-1) and dopachrome tautomerase (DCT) are present in melanosomes and also play a crucial role in catalyzing eumelanin-producing reactions (Hearing et al., 1991; Yasumoto et al., 1997). In mammals, the three melanogenic enzymes are highly similar to metalloproteins. The absence or severe dysfunction of TYRs and other key pigment enzymes results in oculocutaneous albinism (OCA1-4), which presents with intact melanocytes but an inability to make pigment (Lin et al., 2007). Microphthalmia-associated transcription factor (MITF) is the only member of the microphthalmia family of transcription factors known to be essential for melanocyte differentiation and melanogenesis. The regulation of multiple pigmentation-related genes by MITF has solidified the hypothesis that MITF functions as a central regulator of melanogenesis (D'Mello et al., 2016). MITF activity is regulated by a number of signaling pathways. MAPK pathways are known to be involved in melanogenesis by transcriptional or post-transcriptional regulation of MITF (Buscà and Ballotti, 2000). Melanin pigment synthesis occurs in the lysosome-related organelle called melanosome. Melanosomes are typically divided into four maturation stages (I–IV) determined by their structure and the quantity, quality, and arrangement of melanin produced (Kushimoto et al., 2001; Costin et al., 2007). Melanin is packaged and delivered by melanosomes. Melanocyte transfer matured melanosomes of stage IV to neighboring keratinocytes (Orlow, 1995; Slominski et al., 2004). During this process of melanosome transfer, melanocytic transport-related pathways and proteins are activated and involved in melanosome movement from microtubules to actin fiber, which are responsible for melanosome localization at the cell periphery. Meanwhile, the morphology of melanocytes is changed by driving the formation of thinner and longer dendrites (Wu et al., 2001; Fukuda et al., 2002; Kuroda et al., 2003; Westbroek et al., 2003). Therefore, melanin biosynthesis, melanosome formation, maturation, and transfer are crucial to pigmentation, and defects in this process lead to depigmented disorders.

Epimedium brevicornum Maxim. (Epimedii Folium) is a traditional herb widely utilized in China. Epimedium flavonoids are the prominent components in Epimedium brevicornum Maxim. and display pharmacological effects, including promoting bone formation and regulating sexual function. Recent studies have shown that Epimedium flavonoids also play the function of pigment regulation. Icariin, as the main bioactive constituent of Epimedium brevicornum Maxim., has been reported to increase the proliferation of keratinocytes to promote hair growth, as well as promote melanogenic activity and serve as a potent tyrosinase activator (Ye et al., 2010; Su et al., 2017). On the contrary, icariin hydrolysate icariside II and icaritin showed a depigmentation effect, which is associated with inhibition of tyrosinase activity (Park et al., 2008; Zmigrodzka et al., 2018). Since there are melanogenic functional discrepancies between different Epimedium constituents, we are interested in designing comprehensive experiments to uncover the effect of Epimedii Folium extract (EFE) on melanogenesis to obtain a better understanding of the Epimedium herb’s pigmentation pharmacology.

Hence, the aim of our study was to clarify the effect of EFE and its molecular mechanisms on melanin biosynthesis, transport/transfer *in vitro* and *in vivo*. We found that EFE was able to promote the pigmentation process, leading to the synthesis of melanin, and the increase, maturation, and transfer of melanosomes. Specifically, the various trials *in vitro* and *in vivo* provided strong evidence for the pigmentation effects of EFE. Moreover, EFE could significantly promote tyrosinase activity and upregulate the mRNA and protein expression of three key enzymes TYR, TRP-1, and DCT through the p-ERK1/2 signaling pathway by using Western blot, q-PCR, and RNA-sequencing assays. Transmission electron microscopy and immunofluorescence analysis showed that melanosome number, maturation, transport/transfer, and melanocytic dendrite growth/extension were activated and induced by EFE. Finally, we identified the bioactive ingredients in EFE and found that epimedin B (EB) was a high-content, low-toxicity, and effective compound that was responsible for melanogenesis. Our aforementioned data concluded that EFE can target TYRs and the pigment transport/transfer process leading to skin melanogenesis, which might provide a novel rational strategy for the treatment of hypopigmentation and dermatological disorders in pharmaceutical and cosmetic industries.

## Materials and methods

### Chemicals

IBMX (Sigma-Aldrich, China), L-DOPA (Sigma-Aldrich, China), Dulbecco’s modified Eagle medium (DMEM, Gibco, United States), Medium 254 (Gibco, United States), Williams’ Medium E (Gibco, United States), penicillin–streptomycin solution (Beyotime, China), Triton X-100 (BioFroxx, China), BSA (BioFroxx, China), hydroquinone (Aladdin, China), monobenzone (Aladdin, China), animal emulsion cream (Bloomage, China), and other reagents were all purchased from Sinopharm Chemical Reagent (China)

### Herbal material and preparation of Epimedii Folium extract

The leaves of Epimedium brevicornum Maxim. were purchased from Shanghai Kangqiao Chinese Medicine Tablet Co., Ltd. and identified by one of the authors (Yiming Li). A voucher specimen (202007) was deposited at the Department of TCM Chemistry of the School of Pharmacy, Shanghai University of Traditional Chinese Medicine, China.

The leaves of Epimedium brevicornum Maxim. (100 g) were first extracted with 75% ethanol twice for 2 h, concentrated under reduced pressure (27 g) and freeze-dried into a powder, dissolved in DMSO to 40 mg/ml solution, and stored at −20°C. Working concentrations were prepared by diluting with DMEM culture medium.

### Cell and tissue cultures

Cells were cultured in DMEM containing 10% fetal bovine serum and 1% penicillin/streptomycin at 37°C and 5% CO_2_. Primary human melanocytes were cultured in Medium 254 with added human melanocyte growth supplement (HMGS). Normal human/vitiligo skin tissues were cultured in Williams’ Medium E with no fetal bovine serum. The studies on human material were approved by the local ethics committee with Approval Number 2019-735-90-01 in IRB of Shuguang Hospital affiliated with Shanghai University of TCM. Normal human foreskin-derived epidermal melanocytes were derived from young male adult foreskins (ethnic Han/aged 18–22 years). The detailed protocols were according to our team’s published research (Tang et al., 2021).

### Cells viability assay

The viability of B16F10 cells was determined by the CCK-8 kit (Yeasen, China) and MTT kit (Yeasen, China). B16F10 cells (8 × 103/well) were seeded on 96-well plates in DMEM with 10% fetal bovine serum. After adherent growth, fetal bovine serum was decreased to 2.5%, and different concentrations of EFE (1 μg/ml, 5 μg/ml, 10 μg/ml, 20 μg/ml, 30 μg/ml, and 40 μg/ml), epimedin A (1.6 μM, 3.2 μM, 6.3 μM, 12.5 μM, 25 μM, 50 μM, and 100 μM), epimedin B (1.6 μM, 3.2 μM, 6.3 μM, 12.5 μM, 25 μM, 50 μM, and 100 μM), epimedin C (1.6 μM, 3.2 μM, 6.3 μM, 12.5 μM, 25 μM, 50 μM, and 100 μM), icariin (1.3 μM, 2.5 μM, 5 μM, 10 μM, 15 μM, and 20 μM), baohuoside I (1.6 μM, 3.2 μM, 6.3 μM, 12.5 μM, 25 μM, 50 μM, and 100 μM), and icaritin (1.3 μM, 2.5 μM, 5 μM, 10 μM, 15 μM, and 20 μM) were added for 48 h and 72 h; next steps were carried out according to the CCK-8 or MTT kit instructions, and the absorbance was measured at 450 nm and 570 nm using a multi-mode reader (BioTek Instruments, United States).

### Melanin content measurement

Melanin content was measured via a previously described method (Hu, 2008). Cells were treated with isobutylmethylxanthine (IBMX, 100 μM) and EFE (10 μg/ml, 20 μg/ml, 30 μg/ml, and 40 μg/ml) for 72 h [or EFE (40 μg/ml) for 12 h, 24 h, 48 h, and 72 h] and epimedin A (12.5 μM, 25 μM, 50 μM, and 100 μM), epimedin B (12.5 μM, 25 μM, 50 μM, and 100 μM), epimedin C (12.5 μM, 25 μM, 50 μM, and 100 μM), icariin (5 μM, 10 μM, and 20 μM), baohuoside I (1 μM, 3 μM, and 5 μM), and icaritin (0.5 μM, 1 μM, and 2.5 μM) for 72 h. The total melanin in the cell pellet was dissolved in 100 ml of 1 N NaOH/10% DMSO. After incubation for 2 h at 80°C, the solubilized melanin’s absorbance was measured at 405 nm using a microplate reader (BioTek Instruments, United States).

### Cellular tyrosinase activity

Tyrosinase activity was determined by measuring the L-DOPA oxidation rate. The cells were lysed with cell lysis buffer (containing 1% PMSF), and protein content was quantified by a BCA protein assay kit (Beyotime Institute of Biotechnology, China). Then, 100 μl PBS (pH 6.8) containing 20 μg protein was mixed with 100 μl 0.01% g/ml L-DOPA in a 96-well plate. The mixture was incubated at 37°C for 1 h in the dark, and the absorbance was measured at 475 nm (BioTek Instruments, United States).

### Skin tissue staining

Cultured normal human/vitiligo skin tissues were treated with EFE (200 μg/ml for a week) at 37°C and 5% CO_2_. Skin tissues were washed with PBS three times, fixed in 4% paraformaldehyde, and then dehydrated and embedded in paraffin. 5 μm skin sections of were cut with a microtome and stained using a Masson–Fontana dye kit (Sbjbio, BP-DL371, China) and anti-HMB45 (SANTA CRUZ, sc-59305) according to the manufacturer’s instructions. Photos were recorded by microscope (OLYMPUS, BX43F, United States).

Mice dorsal skins were cut off and fixed in 4% paraformaldehyde, then dehydrated, and embedded in paraffin. 5 μm skin sections were cut with a microtome, stained using a standard hematoxylin/eosin procedure, and anti-TRP-1 (Abcam, ab235447) with the corresponding fluorescent secondary antibodies (Servicebio, GB25303), procedures refer to manufacturer’s instructions (Servicebio, China). Photos were recorded by a microscope (OLYMPUS, BX43F, United States).

### Zebrafish experiment

Zebrafish embryos were cultured and selected from the Model Animal Research Center of Nanjing University (Nanjing, China); for the reproduction method, we referred to our team’s published research studies (Tang et al., 2021). PTU was dissolved in water to make stock solutions and then diluted with fresh fish water to 200 mM for all treatments. After treatment with EFE, embryos were collected in 1.5 ml microtubes with 100 μl cell lysis buffer (containing 1% PMSF). Then embryos were ultrasonically crushed for 30 s at 4°C, centrifuged at 12,000 rpm for 10 min, and the sediment was dissolved in 100 μl of 1 N NaOH (containing 10% DMSO) for 2 h at 80°C. The absorbance was measured at 405 nm (BioTek Instruments, United States), and pictures were recorded by Olympus stereoscope and determined by quantitative analysis using ImageJ version 1.8.0.

### Mice experiment

Pursuant to the rules of Laboratory Animal Care and International Law on Animal Experimentation (PZSHUTCM210305006), all animal experiments were executed in agreement with the protocols permitted by the Faculty Animal Committee at the Shanghai University of Traditional Chinese Medicine. Hydroquinone (4% w/w) and EFE (1%, 3% w/w) were dissolved in oil-in-water emulsion cream which contains 61.3% water, 8.0% stearic acid, 8.0% white Vaseline, 7.0% glycerol, 6.0% octadecanol, 5.0% propylene glycol, 2.0% azone, 1.6% trolamine, 1.0% sodium dodecylsulfate, and 0.1% ethylparaben.

Male C57BL/6 mice (6 weeks old) were purchased from Charles River Experimental Animal Technology Co., Ltd. (Shanghai, China). The mice were housed in a controlled environment with a 12-h daylight schedule at a temperature of 23°C ± 1°C and 50% relative humidity with food and water supplied *ad libitum*. After adaptation to the environment for one week, mice were randomized to four groups: control group (only with emulsion cream), model group (with 4% hydroquinone for half day; emulsion cream for half day), EFE low-dose group (with 4% hydroquinone for half day; 1% EFE for half day), and EFE high-dose group (with 4% hydroquinone for half day; 3% EFE for half day [the related study was referred to determine the EFE dose (Su et al., 2017)], five mice in each group. Rosin and paraffin were used to remove mice’s back hair. All mice were applied to back for 45 days and body weight was recorded every 3 days. Pictures were taken to observe the color of hair on day 3 and day 50. Dorsal skins were taken to stain with hematoxylin/eosin and anti-TRP-1 (Abcam, ab235447). Skin tyrosinase activity was measured (the method is the same as that for cellular tyrosinase activity).

### Quantitative real-time polymerase chain reaction

Total RNA was extracted using an RNA isolator (Vazyme, China) and determined with the manufacturer’s protocol. Reverse transcription of RNA into cDNA was performed using the Superscript First-Strand Synthesis System (Thermo Fisher, United States). Real-time polymerase chain reaction (PCR) was carried out on a system (7500 Fast) using SYBR Green PCR Master Mix (Roche Molecular Biochemicals, Germany). Primers sequences, TYR: forward: CAC​CTG​AGG​GAC​CAC​TAT​TAC​G, reverse: GGC​AGT​TCT​ATC​CAT​TGA​TCC​AG; TRP-1: forward: ATC​ATC​GGC​CAA​AAC​GAT​CAT, reverse: GCA​GCT​AAA​ATA​ACA​GGT​GCG​A; DCT: forward: TTC​TGC​TGG​GTT​GTC​TGG​G, reverse: CAC​AGA​TGT​TGG​TTG​CCT​CG; MITF: forward: CAA​ATG​GCA​AAT​ACG​TTA​CCC​G, reverse: CAA​TGC​TCT​TGC​TTC​AGA​CTC​T; β-Actin: forward: GGGAAATCGTGCGTGAC, reverse: AGGCTGGAAAAGAGCCT.

### Western blotting

Cells were lysed with cell lysis buffer (containing 1% PMSF), and protein content was determined using a BCA protein assay kit (Beyotime Institute of Biotechnology, China). The protein was separated by SDS-PAGE, then transferred to a PVDF membrane, and sealed with 5% BSA for 1 h–2 h at room temperature. Anti-TYR (Abcam, ab180753), anti-TRP-1 (Abcam, ab235447), anti-DCT (Abcam, ab221144), anti-MITF (Abcam, ab20663), anti-p-P38/P38 (CST, 4511/8690), anti-p-JNK/JNK (CST, 4668/9252), anti-p-ERK/ERK (CST, 4377/4370), and anti-Actin (Sigma) were incubated at 4°C overnight and then probed with the corresponding second antibody at room temperature for 1 h. The signals were visualized by Tanon 4600SF (Shanghai, China) and determined by quantitative analysis of digital images of gels by using ImageJ version 1.8.0.

### Immunofluorescence staining and transmission electron microscope

Cells were cultured on coverslips in a 12-well plate and treated with EFE (40 μg/ml) for 72 h. Discarding the supernatant, cells were washed with PBS three times, fixed in 4% paraformaldehyde for 10 min and treated with 0.1% Triton for 5 min, and then sealed off with 5% BSA at room temperature for 1 h. Cells were incubated with the primary antibodies, anti-HMB45 (SANTA CRUZ, sc-59305), anti-TRP-1 (Abcam, ab235447), anti-pan cytokeratin (Abcam, ab86734), and FITC-phalloidin (Enzo, 9 A-ALX-350-268-MC01) at 4°C overnight and then incubated with fluorescent secondary antibodies FITC-AffiniPure goat (Yeasen, F2926441) and rhodamine AffiniPure (Yeasen, R1002580) for 1 h at room temperature. Cells were washed with PBS three times and mounted with a mounting medium (with DAPI) (Thermo Fisher, P36935). Photos were recorded by confocal microscope (Leica, SP8).

B16F10 cells were treated with EFE (40 μg/ml) for 72 h and then centrifuged, collected, and fixed in 2.5% glutaraldehyde. The cells were washed with buffer three times for 10 min–20 min, and fixed in 1% osmic acid, dehydrated by gradient ethanol, embedded, and stained. The pictures were recorded by a transmission electron microscope (FEI, Tecnai G2 Spirit BioTWIN).

### RNA transcriptome sequencing

Normal human foreskin-derived epidermal melanocytes were derived from young male adult fore-skins (ethnic Han/aged 18–22 years). The detailed protocols were based on our previous publication (Tang et al., 2021). Primary human melanocytes were treated with EFE (40 μg/ml) for 72 h. Total RNA was isolated for the construction of RNA-seq libraries. The quality of the RNA libraries was evaluated using the Agilent 2200 TapeStation (Agilent Technologies, United States). Library sequencing was performed on a HiSeq 4000 sequencing platform (Illumina Company, United States) by the Novogene Bioinformatics Institute (Beijing, China). Clustering was performed in a cBot cluster generation system using the TruSeq PE Cluster Kit v3-cBot-HS (Illumina). The sequence data were deposited in the BioSample database under the SRA accession number PRJNA862695.

### Separation, analysis, and characterization of Epimedii Folium extract

The EFE profile was obtained in a high-performance liquid chromatography (HPLC) with diode array detector (DAD) system (Agilent 1200). HPLC separation was conducted with an Agilent Zobax SB-C18 column (4.6 mm × 250 mm) maintained at 30°C, a flow rate of 1 ml/min with an injection volume of 10 μl, and detection wavelength was 270 nm. The chromatographic conditions were as follows: the column temperature of 30°C; the detection wavelength used at 270 nm; mobile phase A, acetonitrile; mobile phase B, distilled water. The elution program was set as follows: 0 min–30 min and 24–26% A (v/v); 30 min–35 min and 26–35% A (v/v); 35 min–50 min and 35–50% A (v/v); and 50 min–55 min and 50–52% A (v/v).

EFE was dissolved by ultrasonic with distilled water, separated by C18A31 column chromatography and eluted with a gradient of 25% acetonitrile and 50% acetonitrile to generate two fragments, and analyzed by high-performance liquid chromatography. The elution program was set as follows: 0 min–30 min and 24%–26% A (v/v); 30 min–31 min and 26%–45% A (v/v); 31 min–45 min and 45%–47% A (v/v); 45 min–46 min and 47%–95% A (v/v); and 46 min–60 min and 95%–24% A (v/v). Other conditions are the same as earlier mentioned.

All authentic samples (purity ≥ 95.0%) were purchased from Shanghai Standard Technical Service Co., Ltd. (Shanghai, China).

### Statistical analysis

All data were represented as mean ± SEM. Statistical analysis of results was performed using one-way ANOVA with Dunnett’s multiple comparisons test. All data were analyzed using GraphPad Prism software (United Kingdom). *p* < 0.05, *p* < 0.01, and *p* < 0.001, and all were considered significant.

## Results

### Epimedii Folium extract increases melanin production and promotes tyrosinase activity in a concentration and time-dependent manner

**FIGURE 1:**
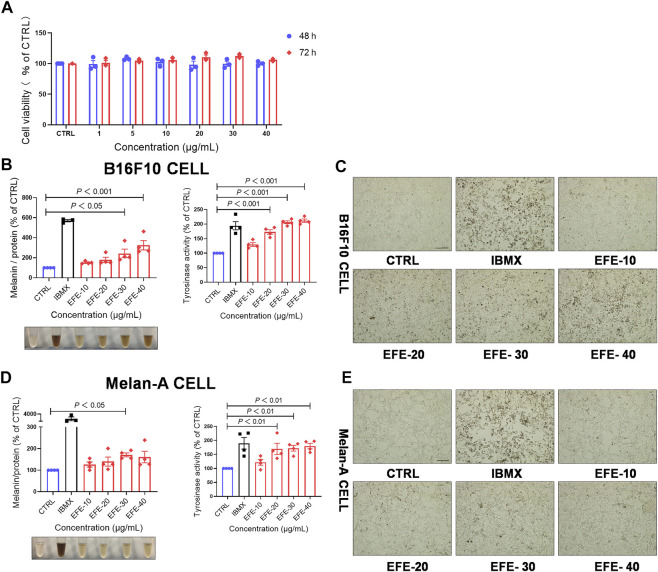
EFE increases melanin production and promotes tyrosinase activity in a concentration- and time-dependent manner. **(A)** CCK-8 cells viability assay (different concentration of 1 μg/ml, 5 μg/ml, 10 μg/ml, 20 μg/ml, 30 μg/ml, and 40 μg/ml for 48 h and 72 h in B16F10 cells); **(B)** melanin content, melanin color, and tyrosinase activity on B16F10 cells based on different concentrations of 10 μg/ml, 20 μg/ml, 30 μg/ml, and 40 μg/ml for 72 h, IBMX (100 μM); **(C)** representative images of intracellular melanin granules on B16F10 cells (scale bar = 100 μm); **(D)** melanin content, melanin color, and tyrosinase activity on Melan-A cells based on different concentration of 10 μg/ml, 20 μg/ml, 30 μg/ml, and 40 μg/ml for 72 h, IBMX (100 μM); and **(E)** representative images of intracellular melanin granules on Melan-A cells (scale bar = 100 μm). For graphical representation, data are presented as mean ± SEM (*n* ≥ 3 in each group), *p* < 0.05, *p* < 0.01, and *p* < 0.001 vs. CTRL are considered significant. Representative image from three independent experiments is shown.

To enrich and acquire the main components in leaves of Epimedium brevicornum Maxim. (Epimedii Folium), we prepared the Epimedii Folium extract (EFE) (for details, refer to materials and methods) for this study. Based on the CCK-8 assay results, 40 μg/ml of EFE showed little cytotoxic effect on B16F10 cells both in 48 h and 72 h (Figure 1A). In the following experiments, the different concentrations of EFE (10 μg/ml, 20 μg/ml, 30 μg/ml, and 40 μg/ml) were selected to observe the melanogenic effect in two melanocyte lines (B16F10 and Melan-A cell) for 72 h. Isobutylmethylxanthine (IBMX), a well-known cAMP-elevating agonist in the melanogenic process, was used as a positive control. Our data showed that intracellular melanin contents were significantly increased in a dose-dependent manner. Notably, EFE increased melanin content by about four times at the highest concentration (40 μg/ml), and the melanin color was visibly darkened (Figure 1B). Consistently, microscopic observation of intracellular substances confirmed greater pigment accumulation along with EFE dose-increasing exposure (Figure 1C). The melanogenesis process is catalyzed by multiple enzymes. Tyrosinase is the pivotal enzyme and its activity determines the velocity of melanogenesis (Slominski et al., 2004). Thus, we tested whether the melanogenic effect of EFF is due to tyrosinase activation by using the oxidation reaction of L-DOPA. Coincident with melanogenesis, tyrosinase activity was clearly promoted in EFE-treated B16F10 cells in a dose-dependent manner (Figure 1B), indicating that EFE could activate tyrosinase to promote melanin synthesis. Additionally, the melanogenic and tyrosinase activity tests were performed on another melanocyte line. Similar effects of melanogenesis and tyrosinase activation were verified in Melan-A cells after EFE treatment (Figures 1D,E). Notably, the melanogenic and tyrosinase activation effects of EFE were abated evidently after being co-treated with tyrosinase inhibitor (hydroquinone) (Supplementary Figure S1A). Thus, all of the aforementioned results co-proved the melanogenic effect of EFE by activating tyrosinase *in vitro*.

**FIGURE 2:**
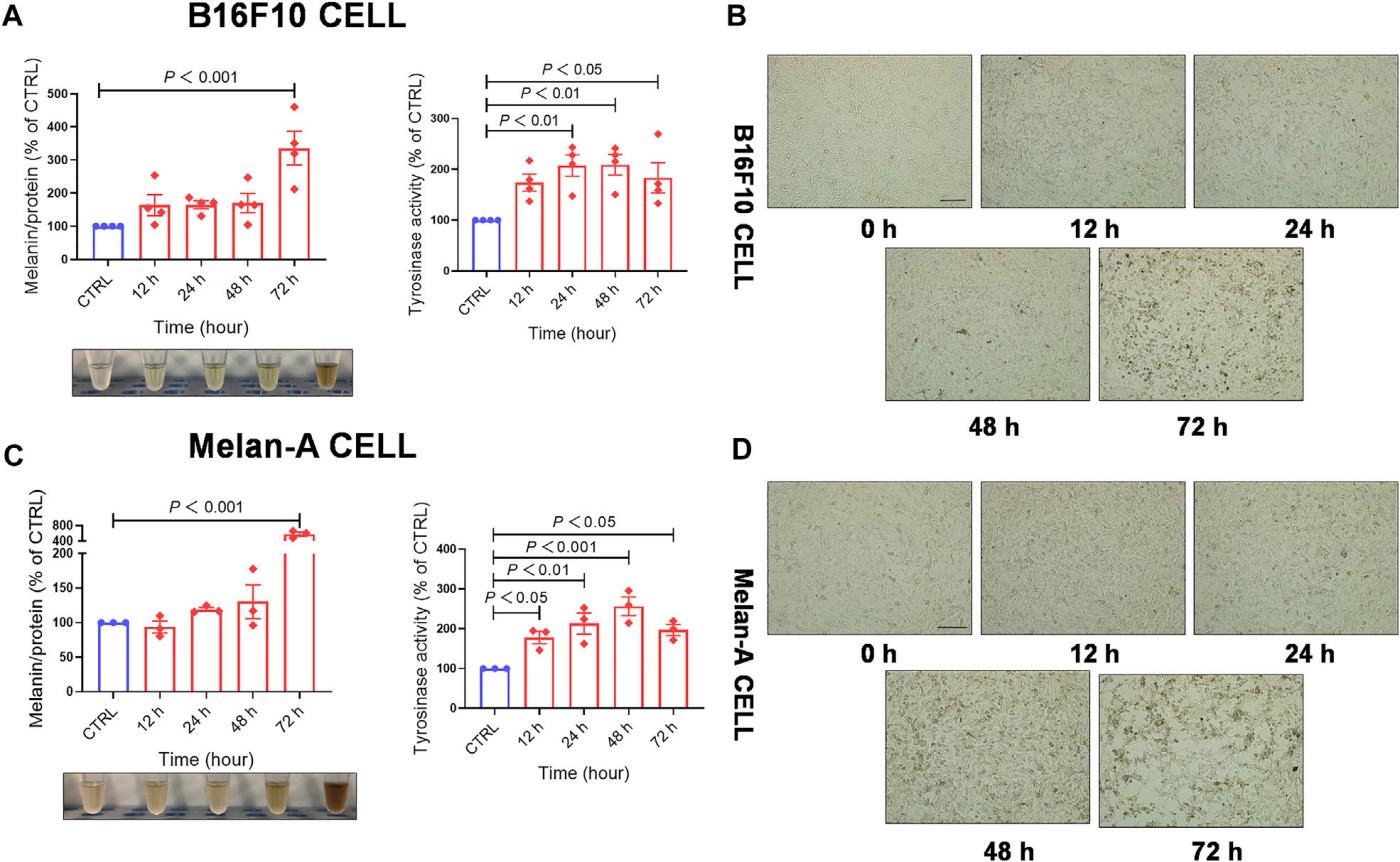
EFE increases melanin production and promotes tyrosinase activity in a concentration- and time-dependent manner. **(A)** Melanin content, melanin color, and tyrosinase activity on B16F10 cells based on different times of 12 h, 24 h, 48 h, and 72 h for 40 μg/ml; **(B)** representative images of intracellular melanin granules on B16F10 cells (scale bar = 100 μm); **(C)** melanin content, melanin color, and tyrosinase activity on Melan-A cells based on different time of 12 h, 24 h, 48 h, and 72 h for 40 μg/ml; **(D)** representative images of intracellular melanin granules on Melan-A cells (scale bar = 100 μm). For graphical representation, data are presented as mean ± SEM (*n* ≥ 3 in each group), *p* < 0.05, *p* < 0.01, and *p* < 0.001 vs. CTRL are considered significant. Representative image from three independent experiments is shown.

To evaluate whether EFE-induced melanogenesis showed a time-dependent manner, melanin contents were examined at different times (12 h, 24 h, 48 h, and 72 h). Interestingly, results revealed that the melanogenic effect of EFE (40 μg/ml) was increased sharply until 72 h, and no obvious changes were observed at other times in both two melanocytes. By contrast, the intracellular tyrosinase activity peaked at 48 h (Figures 2A–D). The reason why tyrosinase activation was evident earlier than melanin content induced by EFE is that the phenotype of melanin production is a progressive process, including melanin synthesis, accumulation, and deposit. However, tyrosinase initiation occurs at an early stage (stage Ⅲ of melanosomes) in the whole melanogenesis bioprocess; thus, there is a time discrepancy between them. To sum up, these results suggest that EFE enhances tyrosinase-catalyzed melanogenesis at 40 μg/ml for 72 h treatment which is chosen in the following experiments.

### Epimedii Folium extract causes pigment-darkening effects in human primary melanocyte, human skin–organ culture, perilesional tissue, and depigmented models of zebrafish and C57BL/6 mice by activating tyrosinase

**FIGURE 3:**
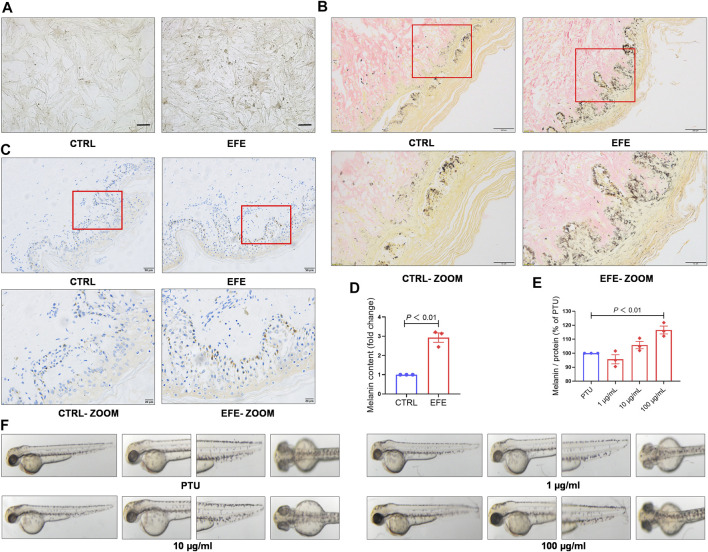
EFE causes pigment-darkening effects in human primary melanocyte, human skin-organ culture, perilesional tissue, and depigmented models of zebrafish and C57BL/6 mice by activating tyrosinase. **(A)** Representative images of HMC under light microscope (scale bar = 50 μm); **(B)** representative images of healthy human skin tissue for Masson–Fontana staining (CTRL, EFE: 200 ×, scale bar = 100 μm; CTRL-ZOOM, EFE-ZOOM: 400 ×, scale bar = 50 μm); **(C)** representative images of perilesional area of vitiligo patients for immunohistochemistry staining (nucleus: blue, melanin granules: brown black; CTRL, EFE: 200×, scale bar = 50 μm; CTRL-ZOOM, EFE-ZOOM: 400 ×, scale bar = 20 μm); **(D)** melanin particle statistics for Masson–Fontana staining; **(E)** melanin particle statistics for zebrafish *in vivo*; **(F)** representative images of melanin in zebrafish. For graphical representation, data are presented as mean ± SEM (*n* ≥ 3 in each group), *p* < 0.05, *p* < 0.01, and *p* < 0.001 vs. CTRL are considered significant. Representative image from three independent experiments is shown.

To further definite melanogenic effect, we cultured human primary melanocytes (HMC). After being treated with EFE (40 μg/ml) for 72 h, HMC secreted more pigment granules under a light microscope (Figure 3A). Cutaneous melanocytes are typically located in the basal layer of the epidermis with many surrounding keratinocytes (Zhang et al., 2020) and transfer their melanin pigment to keratinocytes. In addition to the melanocyte level, we tried to incubate human normal skin tissues with EFE (200 μg/ml) for a week. The treated tissues were stained with the Masson–Fontana method (a melanin-specific dye) to explore the histological pigmentation. As Figures 3B,D, more quantities of melanin signals were produced and distributed under EFE exposure at the junction with the dermis which is featured as the main coexistence of melanocytes and keratinocytes. Hence, these multicellular pigment changes revealed that EFE not only affected melanogenesis in single melanocytes, but also developed the holistic pigmentation efficacy in normal human skin tissue. Due to CD8+T cells infiltration and attack, the melanocytes are absent within the vitiligo lesion, but some are evenly distributed in the basal epidermis of the perilesion region (Riding et al., 2019; Chen et al., 2021). We evaluated EFE repigmented effect in melanocyteless-lesion and perilesion of vitiligo skin by immunohistochemistry analysis. The results showed no apparent positive changes in EFE-induced lesions (Supplementary Figure S1B). Whereas in perilesion, there were more melanosomes (HMB45, a melanosome marker) distributed around the nucleus in the basal epidermis after EFE treatment (200 μg/ml for a week) (Figure 3C). Thus, EFE could cause pigmentation by increasing the melanosome number in perilesion of vitiligo and have less effect in melanocyteless-lesion.

**FIGURE 4:**
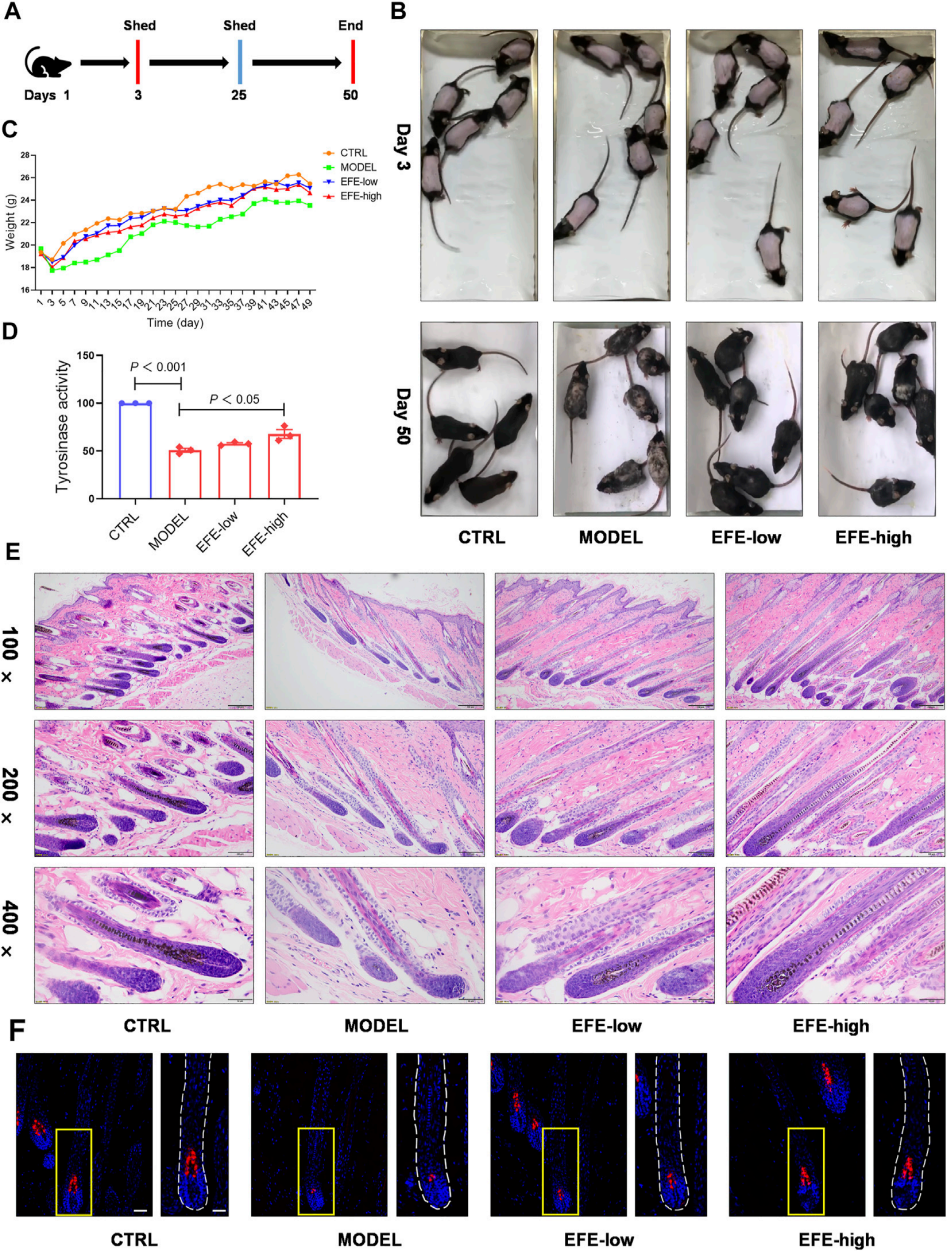
EFE causes pigment-darkening effects in human primary melanocyte, human skin-organ culture, perilesional tissue, and depigmented models of zebrafish and C57BL/6 mice by activating tyrosinase. **(A)** Timeline of mice experiment procedure; **(B)** images of C57BL/6 mice dorsal hair color; **(C)** statistic for mice bodyweight; **(D)** tyrosinase activity of mice dorsal skin; **(E)** representative images of hematoxylin eosin staining of the mice hair follicle (100 ×: scale bar = 200 μm, 200 ×: scale bar = 100 μm, 400 ×: scale bar = 50 μm); **(F)** representative images of immunofluorescence staining of hair follicles (200 ×: scale bar = 100 μm, 400 ×: scale bar = 50 μm). For graphical representation, data are presented as mean ± SEM (*n* ≥ 3 in each group), *p* < 0.05, *p* < 0.01, *p* < 0.001 vs. CTRL are considered significant. Representative image from three independent experiments is shown.

To determine whether EFE can serve as a tyrosinase agonist and have repigmented effect *in vivo*, we developed two kinds of depigmentation models induced by tyrosinase inhibitors in zebrafish and mice, respectively. In the zebrafish model, N-phenylthiourea (PTU) was used as a tyrosinase inhibitor to induce depigmentation. We observed that the depigmented zebrafishes were significantly reversible under 100 μg/ml of EFE for 24 h (Figures 3E,F). Tyrosine is the basic building block of melanin, upon which tyrosinase and other melanogenic enzymes make key modifications. Hydroquinone is frequently used as a depigmenting agent due to its structure which is analogous to tyrosine (Harris, 2017). Thus, we smeared it on the dorsal skin of hairless C57BL/6 mice to interfere with melanogenesis. After about two hair follicle cycles, mice in the control group recovered completely. The only hydroquinone-exposed group presented mass hair graying in mice’s backs. Mice groups treated with EFE (low and high dose) clearly blocked the hydroquinone-induced white-gray hair formation to varying degrees (Figures 4A,B). No obvious difference in weight implied percutaneous safety (Figure 4C). After that, mice dorsal skins were collected and tyrosinase activity was detected. Results showed that EFE could significantly reactivate histological tyrosinase activity in high dose (no statistical difference, but with reactivated effect in low dose) in the hydroquinone-induced mice model (Figure 4D). Thus, the same as *in vitro*, EFE definitely have repigmentation function in two depigmented models by activating tyrosinase *in vivo*. Given that the trait in wild-type C57BL/6 mice, which have black hair but pink skin due to the non-existent melanocytes in the epidermis (Harris et al., 2012), we observed intrafollicular structure where synthesized melanin colors the newly regenerated hair from the root. In control and EFE-treated groups, hematoxylin/eosin staining for longitudinal follicular section presented more pigments in hair bulge and shafts during the anagen stage (growth phase) of the hair cycle, compared to the model group (Figure 4E). In addition, immunofluorescence analysis of TRP-1 (a marker of the melanosome) concomitantly showed that more melanosomes were shaped and distributed at hair follicle bulges induced by EFE (Figure 4F), indicating that the increased number of melanosomes is also a contributor for repigmentation *in vivo*.

Collectively, we used a large methodological spectrum to demonstrate the pigmentation effect of EFE on melanogenesis *in vivo* and *in vitro*, which is initiated by strongly activating tyrosinase and increasing melanosome amount.

### Epimedii Folium extract upregulates the expression of melanogenesis-related genes and proteins through MAPK/ERK1/2 signal pathway for melanogenesis

**FIGURE 5:**
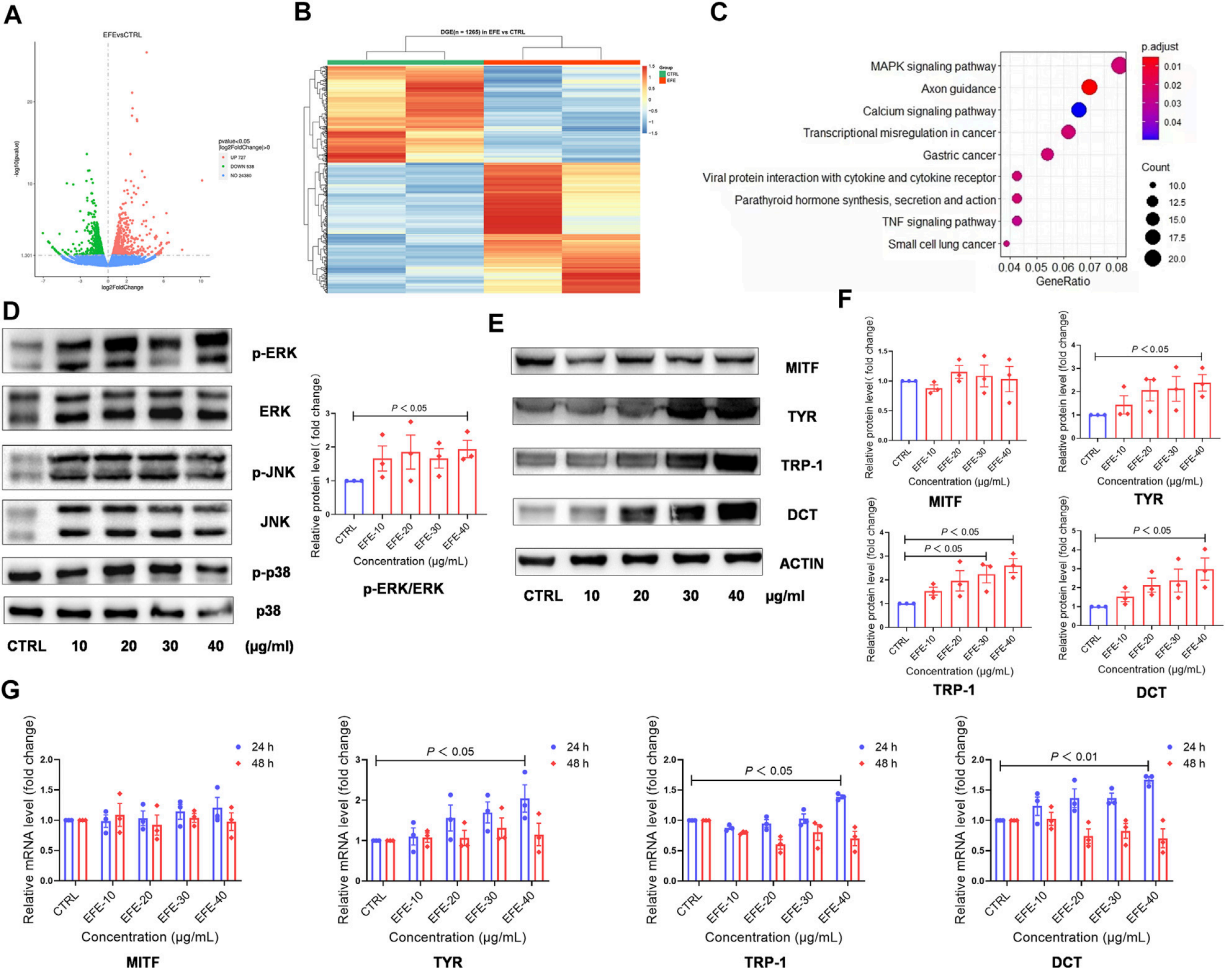
EFE upregulates the expression of melanogenesis-related genes and proteins through the MAPK/ERK1/2 signal pathway for melanogenesis. **(A)** Volcano plots, 727 upregulated differential genes, 538 downregulated differential genes, and 24,380 undifferential genes between CTRL and EFE groups; **(B)** heat map of 1,265 differential genes between CTRL and EFE groups; **(C)** KEGG signal pathways analysis of 1,265 differential genes; **(D)** protein expression levels of p-ERK, ERK, p-JNK, JNK, p-P38, and P38 and statistics for p-ERK/ERK expression (40 μg/ml, 72 h); **(E)** protein expression levels of MITF, TYR, TRP-1, and DCT (40 μg/ml, 72 h); **(F)** statistics for MITF, TYR, TRP-1, and DCT expression; **(G)** mRNA expression levels of MITF, TYR, TRP-1, and DCT gene (40 μg/ml). For graphical representation, transcriptome data are from normal human foreskin-derived epidermal melanocytes, *n* = 2; data are presented as mean ± SEM (*n* ≥ 3 in each group), *p* < 0.05, *p* < 0.01, and *p* < 0.001 vs. CTRL are considered significant. Representative image from three independent experiments is shown.

Based on the previously mentioned EFE-induced visible phenotypic signature, we performed a global transcriptional analysis to delineate the mechanism behind the melanogenic effect of EFE on HMC. Volcano plots and cluster heat map analysis showed that 1,265 significant differential genes (*p*-value ˂ 0.05), including 727 upregulated and 538 downregulated genes, were clustered into a differential hierarchy (Figures 5A,B). We enriched the 1,265 significant differential genes by the Kyoto Encyclopedia of Genes and Genomes (KEGG) analysis, and mass differential genes were shown to converge into the mitogen-activated protein kinase (MAPK) signal pathway (Figure 5C). We further investigated whether EFE activated MAPKs, the classical downstream signaling pathway in melanogenesis responses, in B16F10 cells (D'Mello et al., 2016). As expected, Western blot analysis showed that the phosphorylation of ERK1/2 in the MAPK pathway was significantly enhanced in B16F10 cells, whereas no effect was observed in the phosphorylation of p38 or JNK (Figure 5D, Supplementary Figure S2A). MITF is a well-known pivotal melanogenic master regulator. When MAPK/ERK1/2 is activated, they translocate and regulate transcription factor MITF to trigger further events (Buscà et al., 2000; Slominski et al., 2004; Videira et al., 2013). In Figures 5E,F, our data showed that the expression of MITF protein was not significantly regulated by EFE, which was contradictory to visible phenotypic characteristics. Then, we explored the three major downstream pigment synthesis enzymes: TYR, TRP-1, and DCT. Unsurprisingly, Western blot analysis showed that EFE increased the expression of TYR, TRP-1, and DCT, especially TRP-1 and DCT (Figures 5E,F). To exclude this divergence caused by the post-transcriptional procedure, we checked the expression of MITF and pigment enzymes at the transcriptional level. Consistently, the mRNA expression of pigment enzymes presented the same increasing trend as the proteins for 24 h after EFE treatment, with degradation for 48 h. However, no significant changes in MITF were still measured at the transcription level (Figure 5G). Thus, the intracellular melanogenesis process induced by EFE is achieved by the increased expression of TYR, TRP-1, and DCT through the activation of the ERK1/2 MAPK signaling pathway.

### Epimedii Folium extract raises melanosome number, promotes melanosome maturation/transferring in co-cultured melanocytes and keratinocytes to pigmentation

**FIGURE 6:**
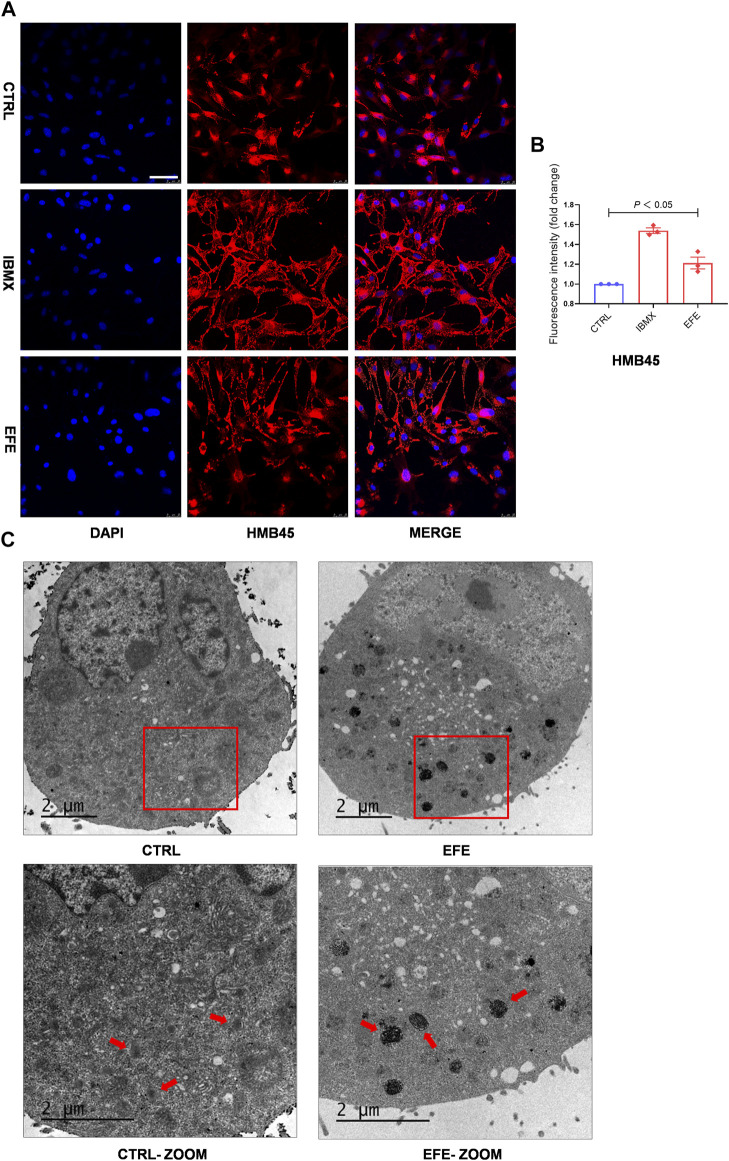
EFE raises melanosome number, promotes melanosome maturation/transferring in co-cultured melanocytes and keratinocytes to pigmentation. **(A)** Representative images of immunofluorescence staining in B16F10 cells (red: HMB45, blue: DAPI; IBMX-100 μM, EFE-40 μg/ml treated for 72 h; scale bar = 50 μm); **(B)** fluorescence intensity statistics for HMB45; **(C)** representative images of melanosome in B16F10 cell using transmission electron microscopy (CTRL, EFE: 6,000 ×; CTRL-ZOOM, EFE-ZOOM: 9900 ×; scale bar = 2 μm). For graphical representation, data are presented as mean ± SEM (*n* ≥ 3 in each group), *p* < 0.05, *p* < 0.01, and *p* < 0.001 vs. CTRL are considered significant. Representative image from three independent experiments is shown.

Melanin granules synthesis occurs predominantly in melanosomes, and melanogenic enzymes are located and worked in the melanosome membrane (Le et al., 2021). Pigmentation differences can arise from variations in the number, composition, and distribution of melanosomes. (Lin and Fisher, 2007). Therefore, it is necessary to investigate more mechanisms of melanogenic melanosome biogenesis caused by EFE. We focused on the melanosome contents that are required for melanin synthesis, the subsequent melanosome ultrastructure and distribution, and then on the melanosome transfer. In Figures 3, 4, the number of melanosomes was preliminarily observed in histological perilesion and mice follicles. For further exploration, we used the confocal fluorescence microscope with melanosome-specific HMB45 antibody to observe the melanosome changes in the interior of melanocytes. The intensity and location of red fluorescence reflected the melanosome number and distribution in B16F10 cells. Fluorescence analysis showed that more melanosomal structures were generated after EFE treatment compared to control. In addition, as to the location detection, red fluorescence in the EFE group was distinctively presented in a sprayed-around pattern, which illustrated the melanosomes were transported and distributed far from the nucleus in melanocyte interiority. IBMX served as a positive control (Figures 6A,B). These results suggest that EFE can not only raise the melanosome number in melanocytes, causing melanin accumulation, but also concurrently promote the melanosomes’ move to the periphery within melanocytes. Early premelanosomes are formed by outpouching of a smooth membrane from the endoplasmic reticulum and then developing into mature melanosomes in four steps (Slominski et al., 2004). Stages I and II are responsible for matrix organization and have few melanin formed in them. In stage III, melanin begins depositing, and in stage IV, melanosomes are fully melanized (completely filled with melanin) (Tian et al., 2021). In melanocytes, only mature melanosomes could be transferred to the cell periphery, whereas immature melanosomes cluster around the perinuclear area, where they originate from. Transmission electron microscopy (TEM) confirmed the fact that an increased number of darker pigmented stage IV melanosomes filled with the fibrillar network were produced in EFE-treated cells (Figure 6C). As a result, EFE positivity regulated melanosome biogenesis, including melanosome contents, melanosome maturation, as well as melanosome movement to the cell periphery in the melanocyte.

**FIGURE 7:**
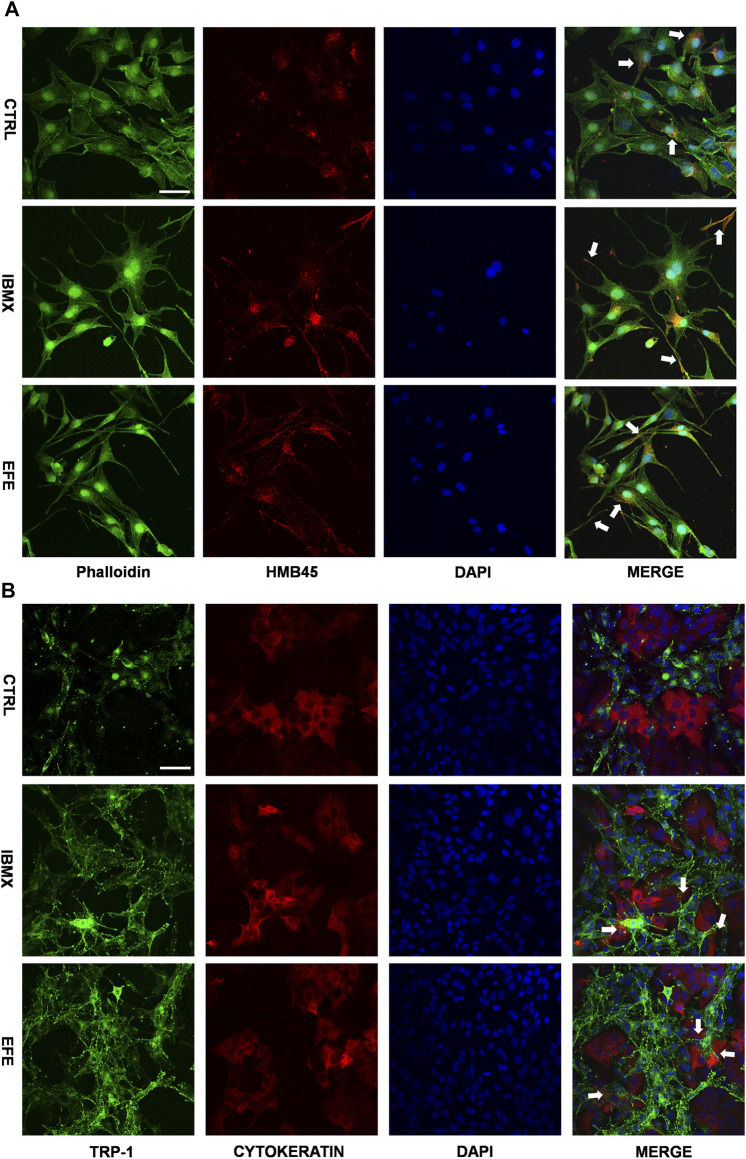
EFE raises melanosome number, promotes melanosome maturation/transferring in co-cultured melanocytes and keratinocytes to pigmentation. **(A)** Representative images of immunofluorescence staining in SK-MEL-28 cells (red: HMB45, green: phalloidin, blue: DAPI; IBMX-100 μM, EFE-40 μg/ml treated for 72 h; scale bar = 50 μm); **(B)** representative images of immunofluorescence staining in B16F10 cells and HaCaT cells co-culture (red: CYTOKERATIN, green: TRP-1, blue: DAPI; IBMX-100 μM, EFE-40 μg/ml treated for 72 h; scale bar = 50 μm). For graphical representation, representative image from three independent experiments is shown.

The scattered effect of matured melanosome distribution indicated intracellular melanin transportation within melanocytes was stimulated by EFE, but this was still unable to account for the pigmentation effect in the epidermis where keratinocytes and melanocytes are about 95% constituents of cell populations (Costin and Hearing, 2007). That is because only intact mature melanosomes transferred from basal melanocytes into adjacent keratinocytes can become melanin dust in the upper layers of the epidermis (Slominski et al., 2004). Thus, this inspired us to examine whether EFE has an influence on melanosome transfer between melanocytes and keratinocytes. Melanocyte dendricity, or dendrite outgrowth, constitutes a very important feature characterizing melanocyte activation, and it is an essential process to ensure melanosomes transfer to keratinocytes (Buscà et al., 2000). To investigate the cellular branch and dendrite alteration, we double-stained the SK-MEL-28 melanocytes with phalloidin antibody (a cytoskeleton marker) to observe dendricity, dendrite morphology, and with HMB45 antibody to detect melanosome number and location. Confocal microscopy analysis showed that EFE morphologically developed more dendricity and longer extension of dendrites, as well as more melanosome number in SK-MEL-28 melanocyte. In particular, more intracellular melanosome signal localizations moved from the perinuclear region to the tip of the dendrites induced by EFE. By contrast, untreated melanocytes were fusiform or polygon-shaped with few dendrites and fewer melanosomes concentrated around the nucleus (Figure 7A). Therefore, the noticeable melanocyte structural dendrite outgrowth and intramelanocytic melanosome transport are promoted and activated by EFE, which are purposed for further melanocyte–keratinocyte transfer. In mammalian melanocytes, mature melanosomes are transported from the perinuclear area, where it originates, to the cell periphery, where they will be further transferred to adjacent keratinocyte (Ross et al., 2008; Tian et al., 2021). The wide variation of human skin color phenotypes is affected by melanin interactive communication between melanocytes and keratinocytes (Benito-Martinez et al., 2021). We co-cultured melanocytes and keratinocytes to assess the melanin transfer effect between them. In co-cultured B16F10 melanocytes marked with TRP-1 (a melanosome maker, green) and HaCaT keratinocytes with cytokeratin (a keratinocyte marker, red) condition, overlapped regions of TRP-1 and cytokeratin were evaluated for intercellular transferring ability by co-localization analysis. As expected, EFE not only raised the melanosome quantity in a single melanocyte (upregulated the expression of TRP-1), but also induced more overlapped fluorescence regions in keratinocytes compared to the control (Figure 7B). In summary, these visualized data suggest that melanocytic dendrite growth/extension, cellular matured melanosome movement, and intercellular melanosome effective delivery programming are largely strengthened, and all of these jointly contribute to the final phenotypic presentation.

### Epimedii Folium extract’s fingerprint; main compounds’ identification; and epimedin B as a high-content, low-toxicity, and effective compound in melanogenesis

**FIGURE 8:**
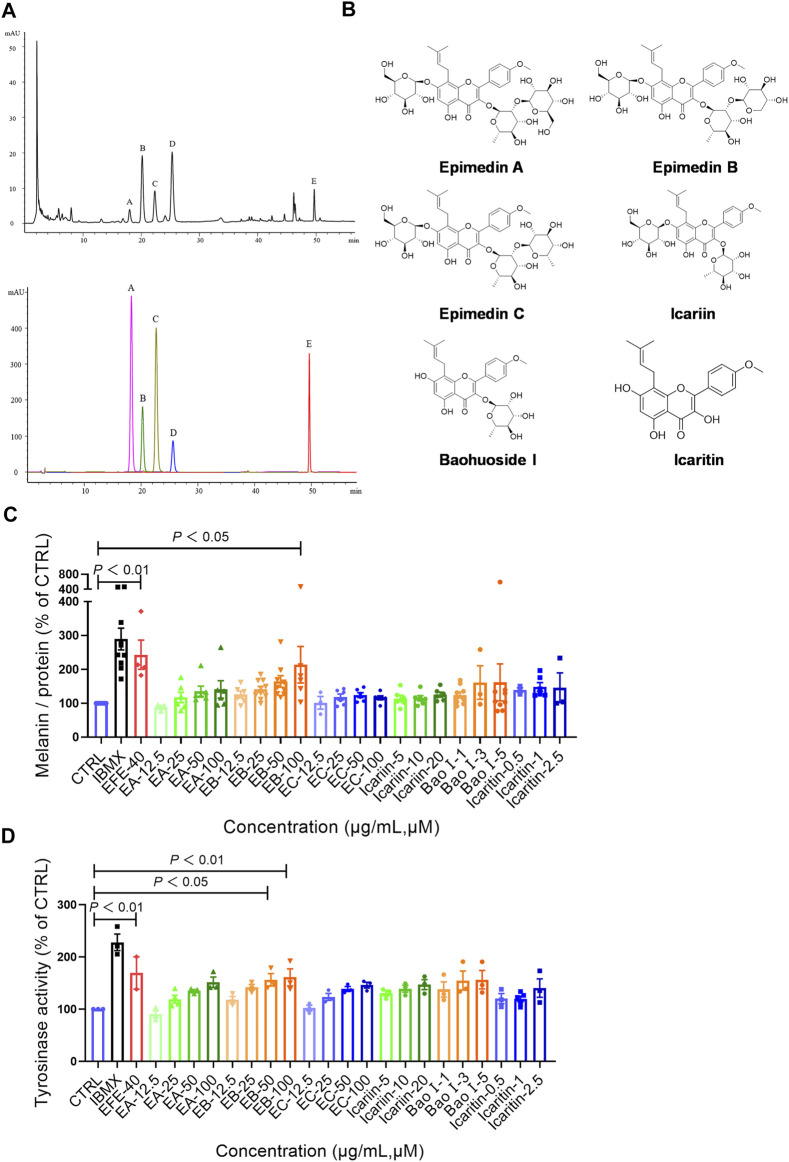
EFE’s fingerprint, main compounds’ identification, and epimedin B as a high-content, low-toxicity, and effective compound in melanogenesis. **(A)** High-performance liquid chromatography profiles of EFE and five main flavonoid components; **(B)** five main components and icaritin structures; **(C)** melanin contents by six components with different concentrations (μM), IBMX (100 μM), and EFE (40 μg/ml); **(D)** tyrosinase activity by six components with different concentrations (μM), IBMX (100 μM), and EFE (40 μg/ml). For graphical representation, data are presented as mean ± SEM (*n* ≥ 3 in each group), *p* < 0.05, *p* < 0.01, and *p* < 0.001 vs. CTRL are considered significant.

**FIGURE 9:**
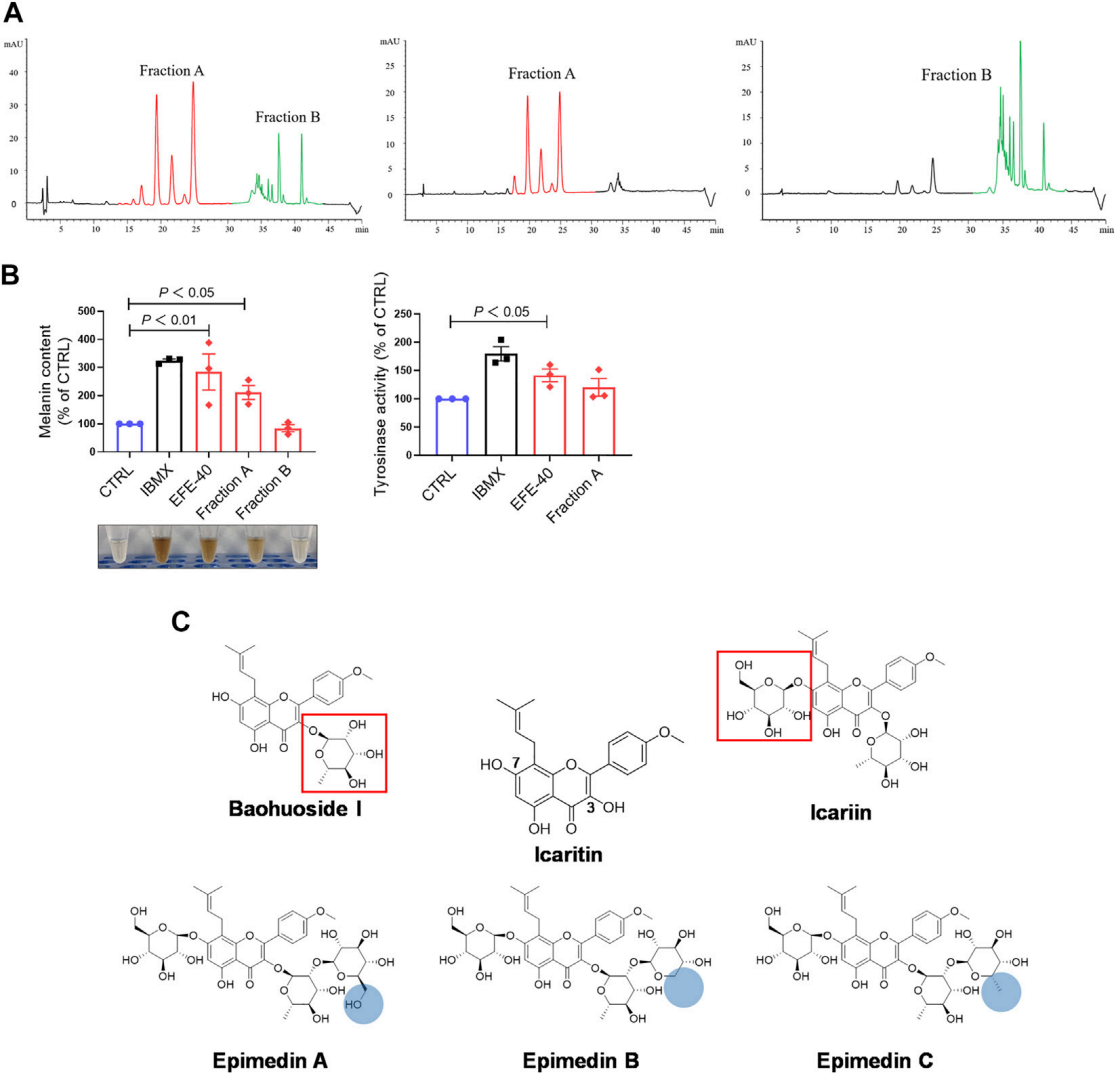
EFE’s fingerprint, main compounds’ identification, and epimedin B as a high-content, low-toxicity, and effective compound in melanogenesis. **(A)** High-performance liquid chromatography profiles of EFE, fraction A, and fraction B; **(B)** melanin content, melanin color, and tyrosinase activity on B16F10 cells (faction A and B: 40 μg/ml, 72 h); **(C)** structural differences of five main components and icaritin. For graphical representation, data are presented as mean ± SEM (*n* ≥ 3 in each group), *p* < 0.05, *p* < 0.01, and *p* < 0.001 vs. CTRL are considered significant.

So far, the effect of EFE on melanin synthesis and melanosome transport/transfer has been investigated based on our studies. Nevertheless, the component that plays the important role in melanogenesis was unknown. To elucidate the melanogenic bioactive constituents in EFE, we used high-performance liquid chromatography (HPLC) to obtain EFE’s fingerprint. Five highest peaks (A–E) were detected and identified by comparing the retention time and ion information with standards. It was confirmed that peaks A–E are epimedin A (A), epimedin B (B), epimedin C (C), icariin (D), and baohuoside I (E), respectively (Figure 8A). Although we did not determine the five components’ exact content in EFE, the two highest components clearly are epimedin B and icariin. Given that compounds A–E belong to glycoside derivatives of icaritin, we incorporated them into the next trials of melanogenic activity (Figure 8B). MTT tests were taken to ensure subsequent experimental concentration was safe and acceptable (Supplementary Figure S2B). Melanin content and tyrosinase activity tests both confirmed that epimedin B (EB) was not only a high-content ingredient, but also an effective melanogenic component in EFE. (Figures 8C,D). In addition, we divided EFE into two fractions depending on component polarity (Figure 9A) and found that fraction A including epimedin A, epimedin B, epimedin C, and icariin showed excellent activity, but fraction B was inactive (the cell toxicity of fraction B is severe; thus, tyrosinase activity is omitted) (Figure 9B). Therefore, the big polar fraction A was the effective melanogenic section of EFE.

## Discussion

In the past studies of Epimedium brevicornum Maxim., almost all of pharmacological research studies focused on bone formation, sexual function regulation, nervous protection, and cardiovascular prevention (Chen et al., 2015). Although some studies of Epimedium flavonoids related to melanogenesis were investigated, the inconsistent outcome was far from satisfactory. In this study, we provided comprehensive evidence of the pigmentation effect *in vivo* and *in vitro*, including melanin synthesis, melanosome increase, maturation, and transfer to keratinocytes, and determined the bases of the main components in EFE for melanogenesis.

Melanins are polymorphous biopolymers that are synthetized by complex multistep transformations of L-tyrosine. During melanin biosynthesis, at least three enzymes (TYR, TRP-1, and DCT) are absolutely required to synthesize different types of melanin, but only TYR is exclusively necessary for melanogenesis (Niu and Aisa, 2017). Hydroxylation of L-tyrosine to L-DOPA is the rate-limiting step in melanin synthesis and is catalyzed by TYR; thus, many drug research studies for skin whitening/blackening all targeted TYR activity (Slominski et al., 2004; Videira et al., 2013; Niu and Aisa, 2017). It has been shown that melanocytes with a low melanin content can synthesize TYR more slowly with higher degradation than melanocytes with a higher melanin content and TYR activity. The trials of two melanocytes and zebrafish/mice tyrosinase-inhibited models *in vitro* and *in vivo* co-confirmed the strong TYR-activated effect induced by EFE (Figures 1, 2, 3E, 4D). This may explain one crucial contributor to the early obligatory and rate-limiting step in the whole melanogenesis process.

After the common obligatory step of tyrosine hydroxylated to dopaquinone, TRP-1 and DCT are further involved in modifying the melanin into different types. TRP-1 and DCT belong to the TYR family of enzymes. In the structure, there are two distinctive melanosomes correlating with the type of melanin produced: elliptical and fibrillar matrix-filled eumelanosomes are responsible for eumelanin, rounded contour, and vesiculoglobular matrix-filled pheomelanosomes for pheomelanin. Interestingly, both eumelanogenic and pheomelanogenic melanosomes can coexist in the same melanocytes, and their ratio determines the skin pigmentation (Slominski et al., 2004). Despite the coexistence of eu- and pheomelanogenic melanosomes, the eumelanin and pheomelanin synthesis pathways diverge. Eumelanogenesis is followed by a series of oxidoreduction reactions with the production of the intermediates dihydroxyindole (DHI) and DHI carboxylic acid (DHICA), which undergo polymerization to form eumelanin. These steps are catalyzed by TRP-1 and DCT, two eumelanogenic enzymes. In contrast, pheomelanogenesis involved conjugation to cysteine or glutathione to transform into pheomelanin (Slominski et al., 2004). Western blot, q-PCR, and immunofluorescence analysis confirmed that EFE can increase TRP-1 and DCT pigment enzyme contents for eumelanogenesis in B16F10 melanocytes and hair follicles of mice (Figures 4F, 5E–G, 7B). Notably, TEM analysis of melanosome’s ultrastructure showed the melanized melanosomes featured more fibrillar matrix and elliptical traits (Figure 6C). This experimental evidence suggests that EFE-promoted eumelanin is a main contributor type for the newly produced mixed pigment.

Unbiased genome-wide transcriptional profiling of EFE-treated HMC revealed that the MAP kinases pathway was a dominant trigger for intracellular melanogenesis (Figures 5A–C). It is well known that MAP kinase signaling molecules have been identified as critical factors for melanocyte development, and interventions in this cascade are effective pigment-regulated targets (Wang et al., 2017). Many studies showed natural products and herbs involved in pigment regulation through the MAPK pathway, including phosphorylated forms of p38, JNK, and ERK1/2 (Wu et al., 2011; Ko et al., 2014; Wang et al., 2017; Kim et al., 2020). Our Western blot analysis showed that only the ERK1/2 phosphorylated form was significantly activated among MAPK cascades after EFE treatment (Figure 5D). MAP kinases ERK1 and ERK2 are serine-threonine kinases that are activated upon phosphorylation by the MAP kinase (MEK). Upon activation, ERKs translocate to the nucleus to activate transcription factors involved in melanocytes cell proliferation or differentiation (Buscà et al., 2000). MITF is a basic helix-loop-helix transcription factor that is mainly expressed in melanocytes, retina pigment cells, mast cells, and osteoclasts (Marks and Walker, 1981; Stechschulte et al., 1987). It is widely accepted that MITF plays a pivotal role in melanocyte development and survival. MITF is shown to specifically bind the M box and E box motifs and to upregulate TYR, TYRP1, and TYRP2 promoter activities (Bertolotto et al., 1998). Western blot and q-PCR assays confirmed that EFE-induced melanogenesis was mediated by upregulating the expression of TYR, TRP-1, and DCT, instead of MITF (Figures 5E–G). Related studies showed that MAP kinases could phosphorylate microphthalmia at serine 73, then MITF phosphorylation regulates MITF association with the transcriptional coactivator p300/CBP to increase its transcriptional activity on the tyrosinase promoter, leading to the stimulation of melanogenesis (Hemesath et al., 1998). Hence, we infer that it is possible that the ERK-dependent phosphorylation of MITF accounts for the up-expression of three downstream enzymes. Intriguingly, the melanogenesis study of the MITF-independent mechanism was also reported in recent years, indicating the probable existence of transcription factors other than MITF (Natarajan et al., 2014). Although the influence on MITF needs further investigation, the EFE-induced melanogenic effect is associated with increased downstream TYRs, which are final melanogenesis executors.

Melanosomes are subcellular special organelles in melanocytes that synthesize melanin pigments. Generally, the number of melanocytes in human skin of all types is essentially constant, the melanosome number, size, and manner determine the pigmentation differences (Slominski et al., 2004). In this study, we observed that EFE could increase melanosome number and regulate the melanosome distribution for pigmentation *in vitro* and *in vivo* (Figures 3C, 4F, 6A,B). Whereas no changes were observed in melanocyteless-lesion of vitiligo, indicating EFE has less effect on melanocyte proliferation (Figure S 1 B). Melanosome biogenesis is a complex process that involved multiple components including the enzymes that catalyze steps in melanin synthesis from tyrosine precursors, solute transporters that allow these enzymes to function, and structural proteins that underlie melanosome shape and melanin deposition (Le et al., 2021). Nascent melanosomes are assembled in the perinuclear region near the Golgi stacks, receiving all enzymatic and structural proteins required for melanogenesis. Melanosomal structural proteins, such as PMEL17 and MART1, are responsible for fiber formation in the early stage melanosomes. In fully striated melanosomes, enzymes such as TYR, TYRP1, and DCT are required to synthesize the phenolic amino acid precursor L-tyrosine into melanin. These synthesized proteins are transported into the melanosome through the endomembrane and cytoskeleton systems (Raposo et al., 2007). Stage I melanosomes are spherical vacuoles lacking TYR activity and have no internal structural components. However, TYR can be detected in the Golgi vesicles, and it has been shown that it is subsequently trafficked to stage II melanosomes. At this point, the presence and correct processing of PMEL17 determine the transformation of stage I melanosomes to elongated, fibrillar organelles known as stage II melanosomes. Melanosomes at stage II contain TYR and exhibit minimal deposition of melanin. After this, melanin synthesis starts, and the pigment is uniformly deposited on the internal fibrils, at which time the melanosomes are termed stage III. The last developmental stage (IV) is detected in highly pigmented melanocytes. These melanosomes are either elliptical or ellipsoidal, electron-opaque due to complete melanization, and have minimal TYR activity. Thereby, the two phenotypes of melanin content and tyrosinase activity caused by EFE in our results can be well-explained based on the melanosome melanized process: the sharp increase in melanin content until 72 h is because melanin synthesis starts at stage III melanosomes for which numerous events of melanosome structural shape and functional enzymes synthesis, transport, traffic have to be performed. The decrease of tyrosinase activity at 72 h is due to the complete melanization of melanosome structure by our TEM result (Figure 6C).

After undergoing the four developmental stages, melanosomes gradually move toward the periphery of the cell within the dendrites (Costin and Hearing, 2007). Melanosome intramelanocyte transport is thought to consist of microtubule-dependent fast transport and actin filament-dependent slow movement (Ross et al., 2008). Thus far, it is still unclear how melanosomes switch from microtubules to actin filaments. Melanocyte dendrites are hormonally responsive actin- and microtubule-containing structures whose primary purpose is to transport melanosomes to the dendrite tips and then transfer them to the neighboring keratinocytes (Tang et al., 2021). Thus, we decided to stain the morphological structure of melanocytes for intuitive dendrites changes and melanosome localization. According to our immunofluorescence analysis, EFE structurally stimulated more dendricity and longer extension of dendrites, and promoted intracellular melanosome movement from the perinuclear region to the dendritic tips (Figure 7A), indicating actin- and microtubule-dependent morphological changes and intracellular transportation are exactly formed and developed.

Once melanosomes reach the plasma membrane following microtubule- and actin-mediated transport, the melanosomes are then transported out of melanocytes to neighboring keratinocytes. Cutaneous pigmentation is the outcome of two important events: the synthesis of melanin by melanocytes and the transfer of melanosomes to surrounding keratinocytes in which pigments are retained to provide the skin and hair with color. So far, the mode of melanosomes transfer has not been clarified clearly, four transferred models were proposed: cytophagocytosis model; membrane fusion model, shedding-phagocytosis model, and exocytosis-endocytosis model (Wu and Hammer, 2014; Tadokoro et al., 2017). To model the interaction between keratinocytes and melanocytes that regulate skin color in physiologic conditions, we co-cultured the melanocytes and keratinocytes. Although we did not explore how melanosomes were transferred from melanocytes to keratinocytes, importantly, the EFE-induced effective transfer process was realized, which is a crucial procedure for skin or hair pigmentation (Figure 7B). Notably, the melanocytes–keratinocytes complex responds to a wide range of environmental stimuli, often in paracrine and/or autocrine manners. Except for the regulation of melanocytes, keratinocytes will secrete cytokines, growth factors, and inflammatory mediators that can increase melanin production and/or stimulate melanin transfer to keratinocytes by melanocytes (Costin and Hearing, 2007). Thus, the coexistent condition of melanocytes–keratinocytes, the effects of melanin synthesis and transfer by EFE are the complex interaction of melanocytes and keratinocytes.

Up to now, more than 130 compounds have been identified in Epimedium species, including flavonoids, icarisides, and other kinds of compounds, such as essential oils, fatty acids, phytosterols, and polysaccharides. Of them, isopentenyl flavonoids such as icariin, epimedin A, epimedin B, epimedin C, and baohuoside I, are the most prominent (Chen et al., 2015). HPLC analysis showed icariin and epimedin B were the most compounds in EFE (Figures 8A,B). Icariin has been regarded as the representative compound and has been reported to show various bioactivities, including melanogenesis and tyrosinase activation in melanocytes (Ye et al., 2010; Wang et al., 2017). In our data, the icariin (20 μM) melanogenic effect was not significantly detected. The reason for the discrepancy is the inconsistent concentration of previous studies. However, icariin with higher concentration showed strong cytotoxicity based on our MTT test (Supplementary Figure S2B). By contrast, epimedin A, epimedin B, and epimedin C are also abundant in Epimedium species, but few bioactivities studies about them are investigated. The melanogenesis and tyrosinase activations of them were shown to different degrees, and EB was the best compound in melanogenesis and tyrosinase activation. In addition, icaritin did not show the inhibition of melanin formation, but neither did clear melanogenesis promotion (inconsistent concentration) (Figures 8C,D). Notably, the twice dose effect of EB (100 μM is equal to 80.8 μg/ml) has a melanogenic effect similar to EFE (40 μg/ml). Thus, it is the complex interactions of multiple chemical compounds, rather than only EB contributing to the final repigmented effect of EFE. But, it also makes sense to identify the representative melanogenic compound or ingredients among EFE, and EB is actually high-content, low-toxicity, and effective melanogenesis compared to other compounds. The bioactive ingredients analysis showed that fraction A was the bioactive section (the great polarity section), and EB was included (Figures 9A,B). Compounds A–E belong to flavonoid glycosides. We searched related studies and found that other flavonoid glycosides such as hyperoside and quercetin derivatives which share a similar structure with EB, showed upregulation of melanogenesis and tyrosinase activity (Niu and Aisa, 2017). Additionally, icaritin, a flavone aglycone of compounds A–E, showed strong cytotoxicity. Whereas the cytotoxicity of the other five compounds was obviously abated when structurally introduced with different sugar moieties at C-3 and C-7 sites (Figure 9C). It is perhaps that glycoside status improves molecule solubility due to the ligand sugar introduction. In addition, what is the active group in the EB molecule? What is the reason for the bioactivity difference among epimedin A, B, and C that is shared with similar structure? We preliminarily infer that the prenyl residue in flavonoid molecules may be responsible for the potent melanogenic activity, and steric hindrance and intermolecular hydrogen bond affect pigment-regulated receptor binding.

## Conclusion

**FIGURE 10:**
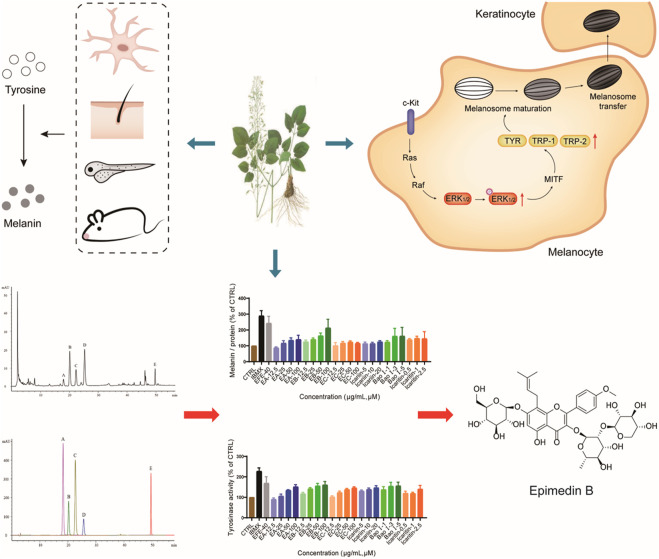
EFE can induce pigmentation *in vivo* and *in vitro* from multi-aspects, including melanin production, melanosome increase, maturation, and transport/transfer to keratinocyte. The melanogenic mechanism is defined by promoting tyrosinase activity, upregulating the expression of tyrosinase families by activating MAPK/ERK1/2 signal pathway. Also, EB is determined as an important melanogenic ingredient.

This study first uncovered and comprehensively investigated the pigmentation effect of the leaf extract of Epimedium brevicornum Maxim. in vitro and *in vivo*. The underlying mechanisms were clarified from two aspects: for the melanin biosynthesis process, the extract promoted tyrosinase activation, and upregulated the expression of TYR, TRP-1, and DCT through the MAPK/ERK1/2 signal pathway; for melanosome biogenesis and transfer procedure, the extract raised melanosome numbers, accelerated melanosome maturation in melanocyte whereby dendrites were stimulated to grow/extend for melanosomes transport, as well as effectively induced melanosome transfer from melanocyte to keratinocyte. Moreover, the six major Epimedium flavonoids were identified, and epimedin B was determined as a high-content, low-toxic, and melanogenic ingredient (Figure 10). Taken together, our results demonstrate that Epimedium brevicornum Maxim. extract induces melanogenesis and melanosome biogenesis, which suggests its novel therapeutic application for hypopigmentation disorders.

## Data Availability

The datasets presented in this study can be found in online repositories. The names of the repository/repositories and accession number(s) can be found below: The sequence data were deposited in the BioSample database under the SRA accession number PRJNA862695.

## Supplementary material

SUPPLEMENTARY FIGURE S1
**(A)** Melanin content, melanin color, and tyrosinase activity on B16F10 cells for 72 h (IBMX, 100 μM; EFE, 40 μg/ml; HQ, 25 μM); **(B)** representative images of lesional area of vitiligo patients for immunohistochemistry staining (nucleus: blue, melanin granules: brown black; CTRL, EFE: 100×, scale bar = 100 μm)

SUPPLEMENTARY FIGURE S2
**(A)** Statistics for p-JNK/JNK, p-p38/p38 expression (40 μg/ml, 72 h); **(B)** MTT tests on five main components and icaritin with different concentrations for 48 h and 72 h. For graphical representation, data are presented as mean ± SEM (*n* ≥ 3 in each group)

## Abbreviations

EFE, Epimedii Folium extract; EB, epimedin B; TYR, tyrosinase; TRP-1, tyrosinase-related protein-1; DCT, dopachrome tautomerase; MITF, microphthalmia-associated transcription factor; IBMX, isobutylmethylxanthine; HMC, human primary melanocytes; PTU, N-phenylthiourea; KEGG, Kyoto Encyclopedia of Genes and Genomes; MAPK, mitogen-activated protein kinase; TEM, transmission electron microscope; HPLC, high-performance liquid chromatography; DHI, dihydroxyindole; DHICA, DHI carboxylic acid.
