# Supplementary figures and images for: Epimedium brevicornum Maxim. Extract exhibits pigmentation by melanin biosynthesis and melanosome biogenesis/transfer
Source: Front Pharmacol. 2022 Sep 29;13:963160. doi: 10.3389/fphar.2022.963160 (PMC9557186; doi:10.3389/fphar.2022.963160)

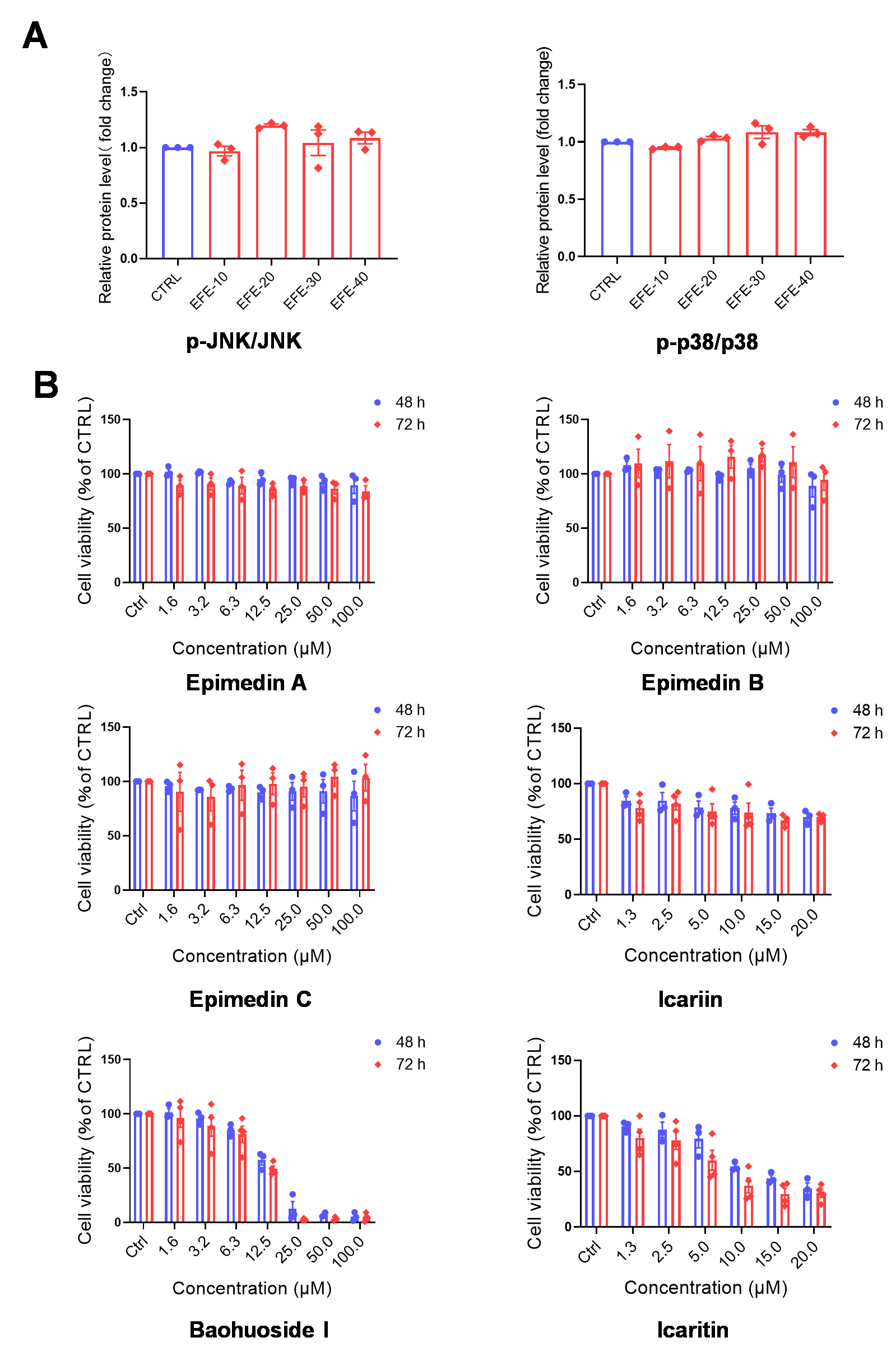

Supplement: Supplementary file 1 [file Image2.TIF]

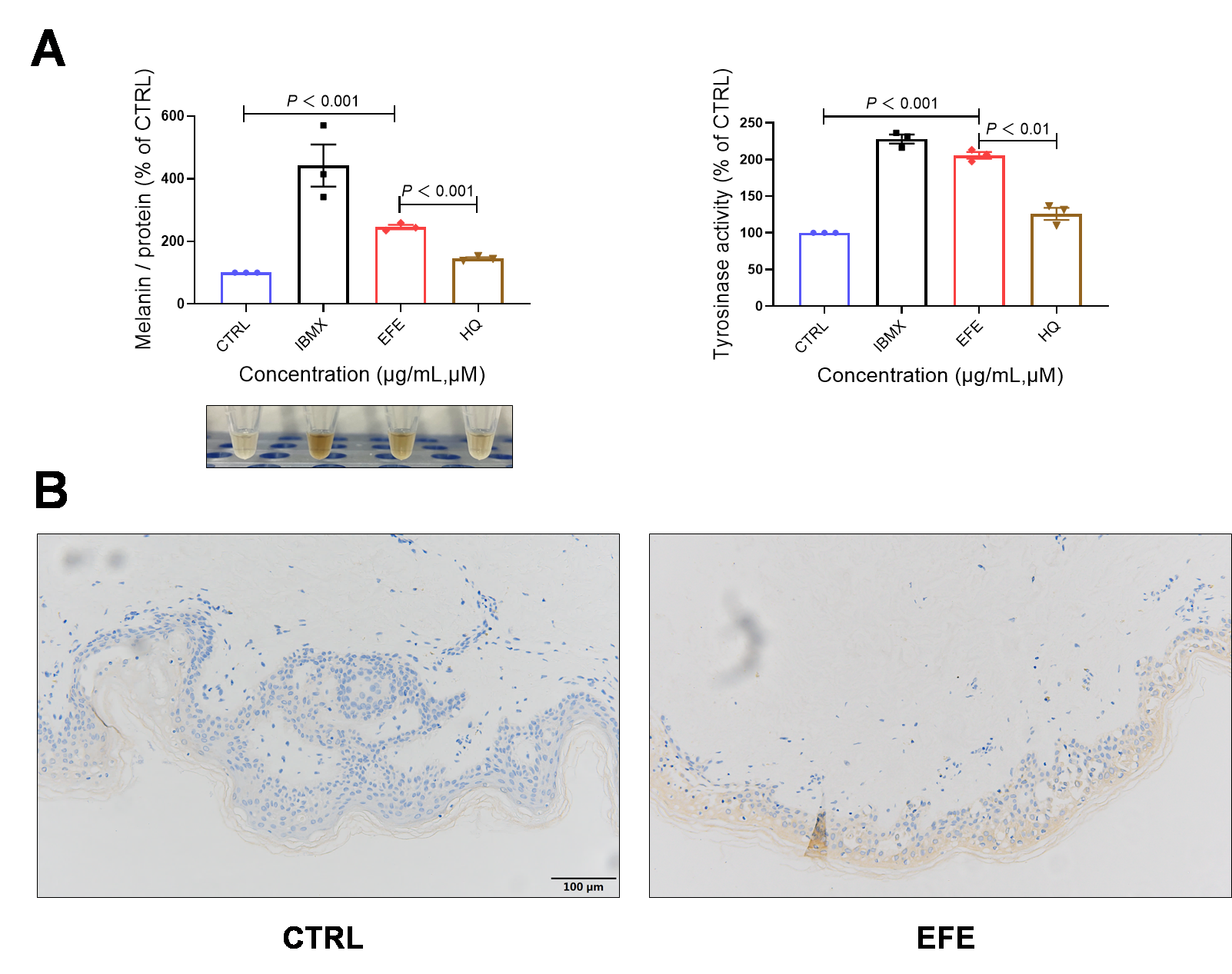

Supplement: Supplementary file 2 [file Image1.TIF]
